# Screening of potential inhibitors targeting the main protease structure of SARS-CoV-2 *via* molecular docking

**DOI:** 10.3389/fphar.2022.962863

**Published:** 2022-10-05

**Authors:** Xinbo Yang, Xianrong Xing, Yirui Liu, Yuanjie Zheng

**Affiliations:** ^1^ School of Information Science and Engineering, Shandong Normal University, Jinan, China; ^2^ Department of Pharmacy, Shandong Medical College, Jinan, China; ^3^ School of Clinical Medicine, Harbin Medical University, Daqing, China

**Keywords:** SARS-CoV-2, main protease, molecular docking, virtual screening, drug repurposing, molecular dynamics

## Abstract

The novel coronavirus disease (COVID-19) caused by SARS-CoV-2 virus spreads rapidly to become a global pandemic. Researchers have been working to develop specific drugs to treat COVID-19. The main protease (M^pro^) of SARS-CoV-2 virus plays a pivotal role in mediating viral replication and transcription, which makes it a potential therapeutic drug target against COVID-19. In this study, a virtual drug screening method based on the M^pro^ structure (Protein Data Bank ID: 6LU7) was proposed, and 8,820 compounds collected from the DrugBank database were used for molecular docking and virtual screening. A data set containing 1,545 drug molecules, derived from compounds with a low binding free energy score in the docking experiment, was established. N-1H-Indazol-5-yl-2-(6-methylpyridin-2-yl)quinazolin-4-amine, ergotamine, antrafenine, dihydroergotamine, and phthalocyanine outperformed the other compounds in binding conformation and binding free energy over the N3 inhibitor in the crystal structure. The bioactivity and ADMET properties of these five compounds were further investigated. These experimental results for five compounds suggested that they were potential therapeutics to be developed for clinical trials. To further verify the results of molecular docking, we also carried out molecular dynamics (MD) simulations on the complexes formed by the five compounds and M^pro^. The five complexes showed stable affinity in terms of root mean square distance (RMSD), root mean square fluctuation (RMSF), radius of gyration (Rg), and hydrogen bond. It was further confirmed that the five compounds had potential inhibitory effects on SARS-CoV-2 M^pro^.

## 1 Introduction

From December 2019, the world witnessed an outbreak of an acute respiratory disease (Han et al., 2020; Rothan and Byrareddy, 2020). In the early stages of the disease outbreak, Zhou et al. (2020) obtained the full-length genomic sequences of the virus collected from five patients. These sequences exhibited 79.6% homology with SARS-CoV. In addition, the newly found virus exhibited 96% identity to bat coronavirus at the whole-genome level. The International Committee of Taxonomy of Viruses named the virus “severe acute respiratory syndrome coronavirus 2” (SARS-CoV-2), and the World Health Organization (WHO) announced this new disease as a novel coronavirus disease 2019 (COVID-19) (Anand et al., 2020; Wang et al., 2020). According to data from the WHO, over 526 million confirmed cases and over six million deaths have been recorded by 29 May 2022.

Similar to SARS-CoV, SARS-CoV-2 also belongs to the *β*-coronavirus class but is more contagious and mutable (Tang B. et al., 2020; Shereen et al., 2020). Vaccination has been widely promoted as an important preventive measure against COVID-19. As on 9 September 2022, the WHO reported that there were 371 COVID-19 vaccine candidates in development, of which 172 have entered clinical trials (COVID-19 vaccine tracker and landscape, 2022, https://www.who.int/publications/m/item/draft-landscape-of-covid-19-candidate-vaccines). Among the vaccines in clinical development, the number of types ranked was protein subunit vaccines (32%), RNA vaccines (23%), viral vector (non-replicating) vaccines (13%), inactivated virus vaccines (13%), DNA vaccines (9%), and other types of vaccines. As research on protein subunit vaccines was relatively mature and was the priority vaccine development method, the number of protein subunit vaccines was the largest among COVID-19 vaccines. However, persistent mutations of the virus can affect the vaccine’s preventive effect, especially Omicron, which largely evaded the antibodies elicited by the vaccine (Planas et al., 2022). SARS-CoV-2 comprises a single-stranded positive-sense RNA genome that encodes both structural and non-structural proteins. The non-structural proteins include RNA-dependent RNA polymerase, coronavirus main protease (M^pro^, also known as 3C-like protease, 3CL^pro^), and papain-like protease (PL^pro^). When the viral genome enters the host cell, the host cell protein translation mechanism translates it into a viral polyprotein, which is then cleaved into effector proteins by the viral proteases M^pro^ and PL^pro^ (Tang X. et al., 2020; Liu et al., 2020; Zhang et al., 2020). Since M^pro^ can cleave polyproteins at no less than 11 conserved sites, it plays a vital role in the replication of viral particles (ul Qamar et al., 2020). Therefore, it is an attractive target for the screening of antiviral inhibitors. The high-resolution crystal structure of SARS-CoV-2 M^pro^ was presented by the Zihe Rao and Haitao Yang’s research team. They also provided a basis for drug screening and design based on the structure of the M^pro^ (Jin et al., 2020).

The research and development of a new drug is a time-consuming process that requires huge financial investment. In the current global crisis, the repositioning of existing drugs seems to be a potentially useful tool in searching for new therapeutic options (Serafin et al., 2020). Computer-assisted virtual screening provides an inexpensive and rapid alternative to high-throughput screening for drug discovery. Furthermore, virtual screening technology can optimize the selection of potential drugs (de Carvalho Gallo et al., 2018). In the past few decades, virtual screening has played an important role in the discovery of small molecule inhibitors of therapeutic targets. Various ligands and structure-based virtual screening methods have been used to identify small-molecule ligands for proteins of interest (Bharatham et al., 2017; Singh and Jana, 2017; Li et al., 2020). Virtual screening technology has revealed several compound molecules that can inhibit SARS-CoV activity (Wei et al., 2006; Niu et al., 2008; Wang et al., 2017).

In this study, we investigated potential M^pro^ inhibitors using a docking-based virtual screening approach. We used a variety of screening strategies, such as molecular docking, molecular dynamics (MD) simulations, biological activity, and ADMET prediction. The AutoDock Tools were used to prepare the M^pro^ receptor model of SARS-CoV-2. A Vina-based molecular docking program was encoded, and M^pro^ and compounds (from DrugBank, with the 3D structure) were docked. The compounds were sorted based on the combined free energy score. The potential drug compounds with inhibitory effects on M^pro^ were determined by analyzing the binding mode between the compounds with better scoring results and M^pro^. The bioactivity and ADMET properties of the five selected compounds were further explored. Simultaneously, we performed MD simulation experiments on the complexes of five compounds and M^pro^. The purpose of this study was to identify potential drug compounds from DrugBank by molecular docking and MD simulations. This method can rapidly predict whether a compound has inhibitory effect on the activity of M^pro^ based on the physicochemical properties of the compound and the stability of the protein–ligand complex.

## 2 Materials and methods

### 2.1 Receptor (SARS-CoV-2 M^pro^ protein) preparation

SARS-CoV-2 M^pro^ is a key CoV enzyme, which plays a pivotal role in mediating viral replication and transcription, making it an attractive drug target for treating COVID-19 (Anand et al., 2002; Yang et al., 2003; Jin et al., 2020).

**FIGURE 1:**
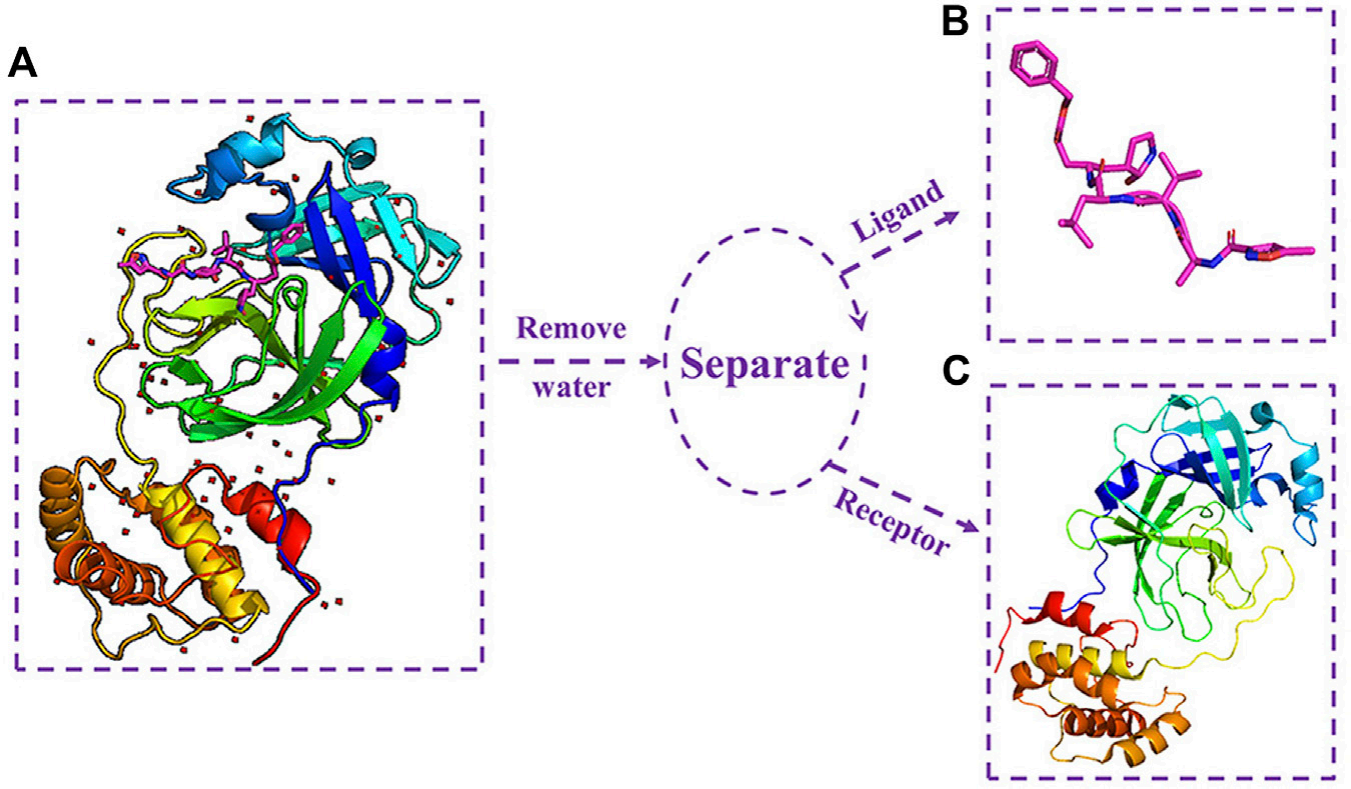
**(A)** Complex crystal structure of M^pro^ protein with N3; **(B)** three-dimensional structure of N3 inhibitor; and **(C)** three-dimensional structure of M^pro^ protein.

The complex crystal structure of M^pro^ and the N3 inhibitor (PDB ID: 6LU7) (Jin et al., 2020) was downloaded from the Protein Data Bank (http://www.rcsb.org). M^pro^ was isolated from the complex crystal structure using PyMOL. The separation process of M^pro^ and N3 inhibitor is shown in Figure 1. Figure 1A shows the complex crystal structure of M^pro^ protein with N3, and (c) shows the 3D structure of M^pro^.

### 2.2 Ligand data set preparation

For the docking experimental ligand (which is composed of a drug molecule data set and N3 inhibitor), the N3 inhibitor was isolated from the SARS-CoV-2 main protease crystal complex. Figure 1B shows the 3D structure of N3 inhibitor.

The drug molecule data set contained 8,820 molecules with their 3D structures. They were obtained from DrugBank (https://www.drugbank.ca/) in the SDF format (Wishart et al., 2018).

### 2.3 Pre-processing of receptor and ligands

The docking program requires files stored in the Protein Data Bank, especially in the Partial Charge and Atom Type (PDBQT) format. M^pro^ standardization involved Gasteiger charges and the addition of polar hydrogen atoms. The conversion of the file format from the Protein Data Bank (PDB) format to PDBQT format was implemented using AutoDock Tools.

Data standardization was performed as a part of the pre-processing. The drug molecules of the ligand data set were first added to polar hydrogen atoms using Open Babel software (O’Boyle et al., 2011). Subsequently, Gasteiger charges were added using Raccoon (Forli et al., 2016) and broken down into 8,820 small molecule files in a PDBQT format.For the N3 inhibitor, Open Babel software was used to add polar hydrogen atoms and Gasteiger charges, followed by converting the format from PDB to PDBQT.

### 2.4 Molecular docking and screening

**FIGURE 2:**
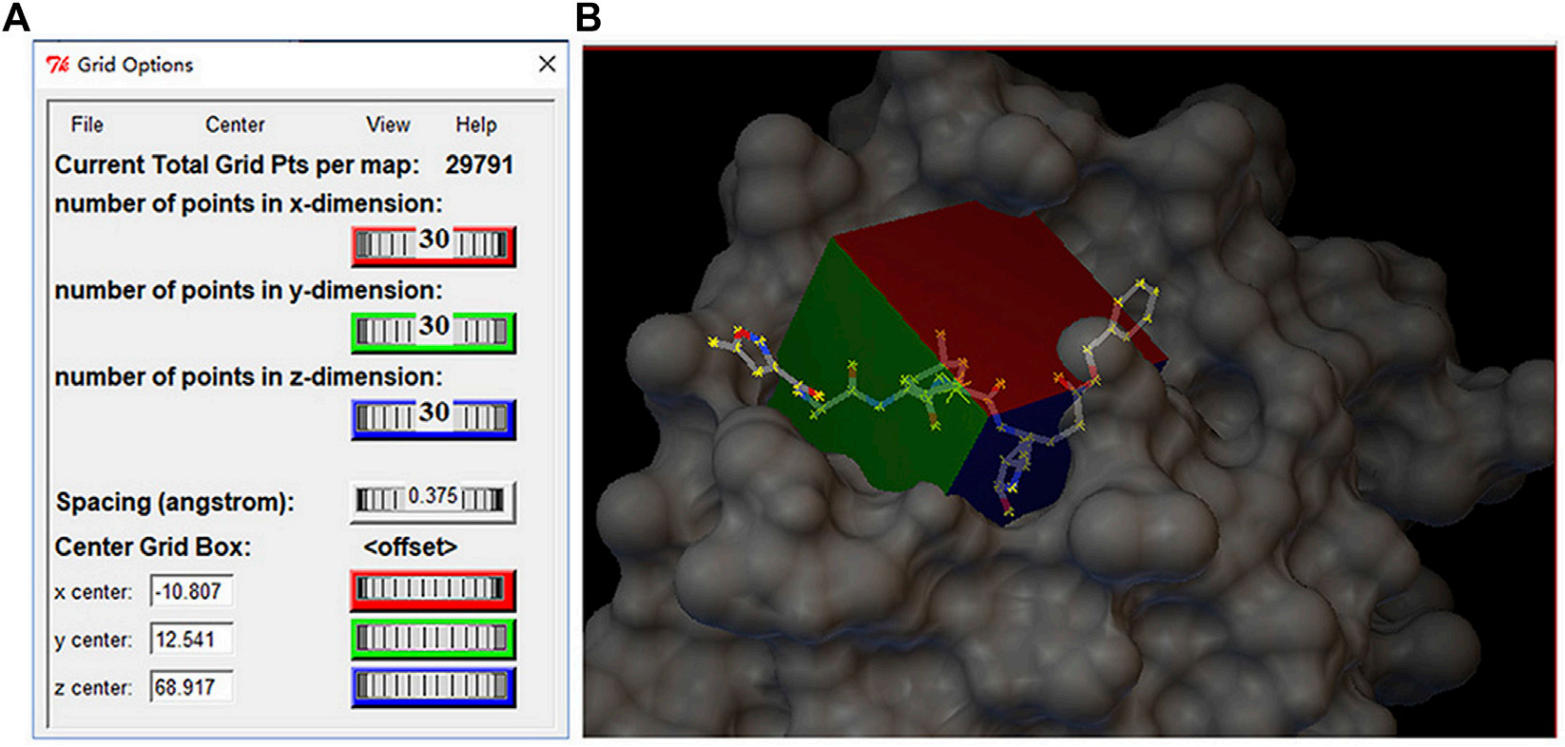
**(A)** Setting information of the grid box and**(B)** three-dimensional structure of gird box in the M^pro^ protein.

Molecular docking was performed using AutoDock Vina and the standardized docking data. In this study, the center of grid box was set to (–10.807, 12.541, 68.917) Å for (center_x, center_y, center_z). Meanwhile, the size of the grid box was defined as (30, 30, 30) Å for (size_x, size_y, size_z). Figure 2A shows the setting information of the grid box, and (b) shows the 3D structure of the grid box for M^pro^. To generate as many different binding modes as possible, the num-modes was set to 20 (maximum number of binding modes to generate), and the energy range was set to 6 kcal/mol (maximum energy difference between the best binding mode and the worst one displayed [kcal/mol]). The number of CPUs was set to 20 (CPU = 20), and the explicit random seed was set to 200.

We encoded a bash script file to implement the docking process. This script file encapsulated the Vina program and the parameters required for the Vina program, including the parameters set in the previous paragraph and the input and output parameters. It could automatically execute the Vina program and perform docking experiments with each ligand molecule and the receptor and finally showed the score of each ligand molecule. It was used to calculate the free energy score of M^pro^ with different conformations of each ligand.

**FIGURE 3:**
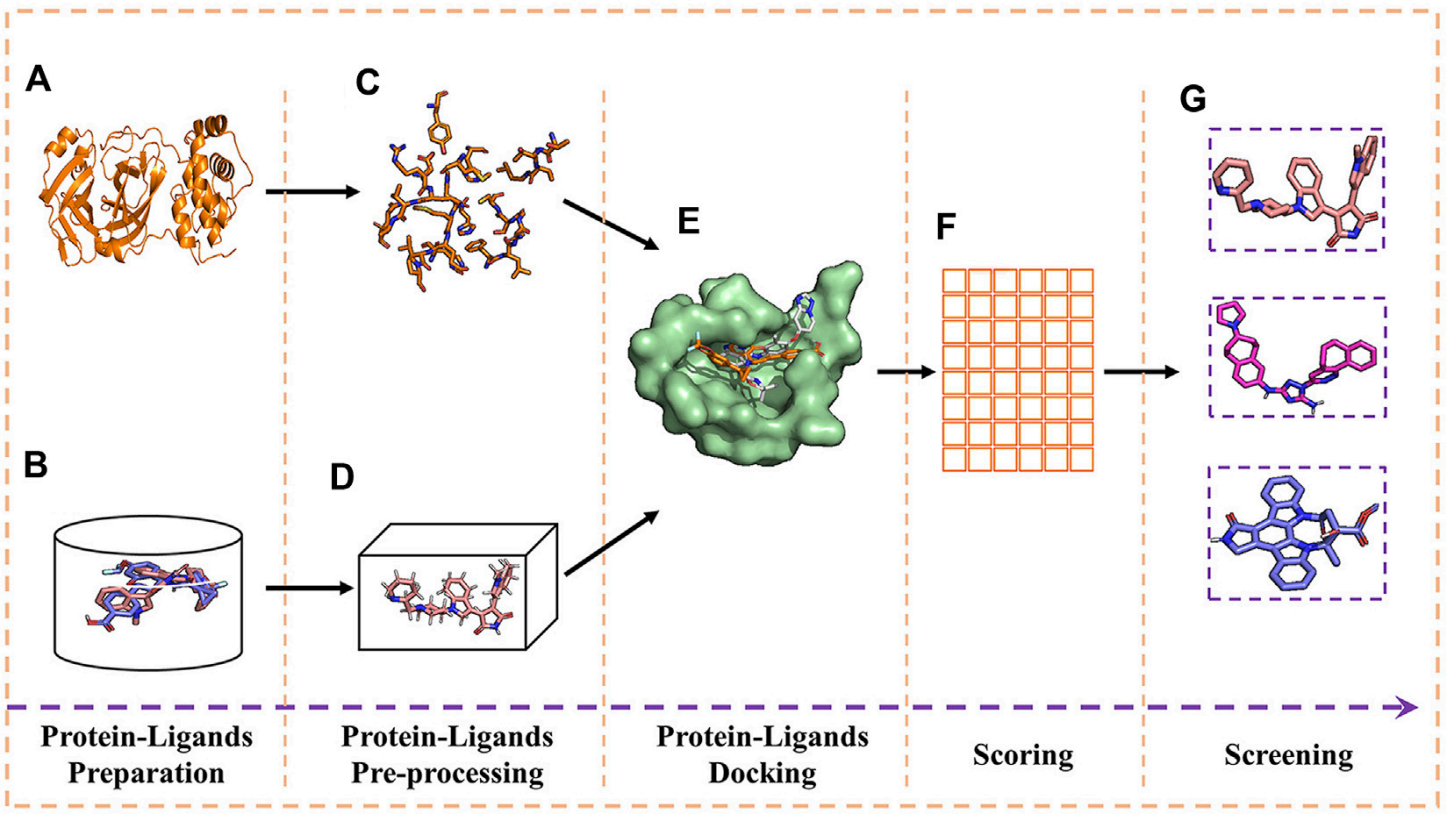
Experimental process of molecular docking and virtual screening. **(A)** Receptor; **(B)** ligands; **(C)** receptor pre-processing; **(D)** ligand pre-processing; **(E)** docking of receptor and ligands; **(F)** table of free energy score; and **(G)** virtual screening for the docking result.

Screening for the potential drug molecule was achieved by implementing a specific Python script program. The optimal docking score of M^pro^ with the N3 inhibitor was used as a reference standard for the analysis and screening to establish a candidate drug molecule data set. Figure 3 shows the experimental process of molecular docking and virtual screening.

Discovery Studio Visualizer was used to analyze the interactions and types of interactions between compounds and M^pro^ (docking complexes).

### 2.5 Molecular dynamics simulation

Molecular dynamics (MD) simulation is an effective method to predict the stability of protein–ligand complexes (Bharadwaj et al., 2021). In this study, we used GROMACS (version: 2022.2, https://www.gromacs.org/) for molecular dynamics simulation. The topology file for M^pro^ protease was generated using the gmx pdb2gmx command, with the addition of the gromos53a6 force field. The topologies of the five drug molecules were generated using the PRODRG (http://davapc1.bioch.dundee.ac.uk/) server, and their respective topology files with parameters set to chirality: Yes; charge: Full; and EM: NO was also generated.

The simulation system adopted a rectangular solvated box and used gmx grompp for the energy detection and minimization processes. The maximum number of minimization steps to perform was set to 50,000, the energy step size was set to 0.01, and the energy minimization algorithm adopted the steepest descent minimization. At the same time, the system was stabilized by 100 ps NVT and NPT balance. The V-rescale thermal bath coupling algorithm was used in the NVT ensemble, and the Parrinello–Rahman pressure coupling method was used in the NPT ensemble. Finally, we performed 100 ns MD simulations of the equilibrium system at a temperature of 300 K and pressure of 1 bar. The RMSD, RMSF, Rg, number of hydrogen bonds, and protein–ligand interactions of the MD simulation results were recorded and analyzed for further validation of our virtual screening results. The results of molecular dynamics simulations were visualized using qtgrace (version: V26) software.

### 2.6 Bioactivity and ADMET property prediction

As an alternative to clinical experiments, computer technology was a fast and efficient method to predict the pharmacodynamic properties of compounds (Zaki et al., 2022). We combined the DrugBank database and used Molinspiration Cheminformatics (https://www.molinspiration.com) and admetSAR web service (Yang et al., 2019) to predict the bioactivity and ADMET properties of the five screened compounds, respectively.

## 3 Results

### 3.1 Molecular docking and screening

In this study, AutoDock Vina was used to perform the docking of the screened molecules with modeled M^pro^. Each ligand generated 20 conformations. These conformations were further subjected to virtual screening evaluation. From the docking search, the conformation with the lowest docked energy was selected as the best conformation. The molecular docking results for AutoDock Vina are presented in Supplementary Table S1.

**FIGURE 4:**
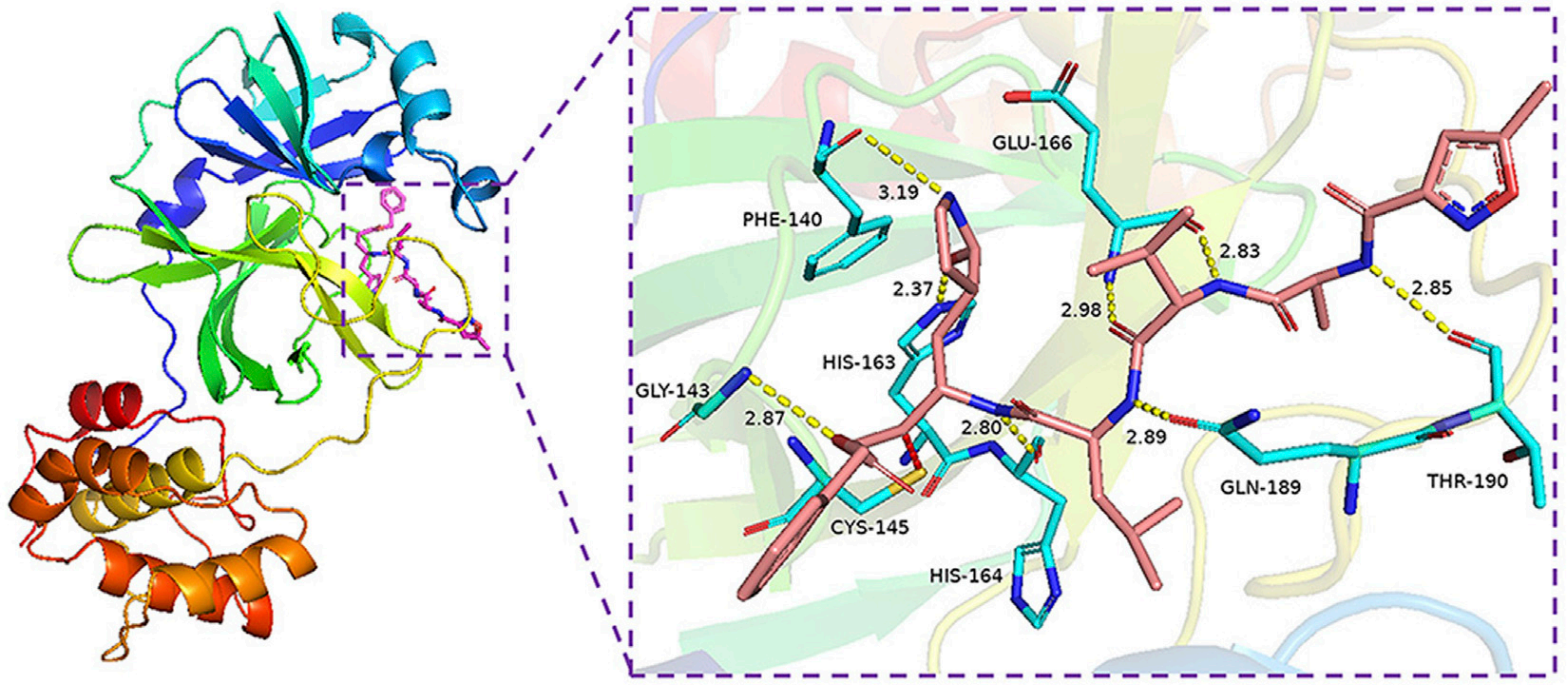
Interaction of covalent bonding and hydrogen bonding between M^pro^ protein and N3.

The binding energy of the interaction of N3 with M^pro^ was -7.8 kcal/mol. N3 is mainly stabilized by interacting with M^pro^ through the formation of covalent and hydrogen bonds. The S atom of Cys145 of M^pro^ forms a covalent bond with C20 of N3. As shown in Figure 4, N3 forms seven hydrogen bonds with Gly143, His164, Glu166, Thr190, Gln189, His163, and Phe140 of M^pro^ (Tang B. et al., 2020). These results show the active pocket position of M^pro^. As a reference, 1,545 compounds, with energy values lower than -7.8 kcal/mol, were obtained. The groups of these compounds in DrugBank are listed in Table 1. According to the order of energy value, the top 30 compounds from the molecular docking analysis are listed in Table 2.

**TABLE 1:** Groups of 1,545 compounds in DrugBank.

No.	Group	Count
1	Approved	95
2	Approved; experimental	4
3	Approved; experimental; investigational	1
4	Approved; investigational	108
5	Experimental	736
6	Experimental; investigational	8
7	Investigational	543
8	Others	50

**TABLE 2:** Top 30 compounds from the docking results.

No.	Accession number	Chemical formula	Group	Binding energy (kcal/mol)
1	DB12983	C_32_H_18_N_8_	Investigational	-10.7
2	DB12225	C_36_H_45_N_5_O_5_S	Investigational	-10.4
3	DB11651	C_30_H_23_N_5_O	Investigational	-10.3
4	DB13050	C_38_H_52_N_6_O_2_	Investigational	-10.2
5	DB11913	C_28_H_25_FN_6_O_3_	Investigational	-10.1
6	DB14883	C_29_H_24_FN_7_O	Investigational	-10.1
7	DB14894	C_28_H_21_F_4_NO_7_	Investigational	-10.0
8	DB00320	C_33_H_37_N_5_O_5_	Approved; investigational	-9.9
9	DB06486	C_32_H_29_N_5_O_2_	Investigational	-9.9
10	DB08450	C_21_H_16_N_6_	Experimental	-9.8
11	DB12411	C_30_H_34_N_8_	Investigational	-9.8
12	DB00696	C_33_H_35_N_5_O_5_	Approved	-9.7
13	DB04868	C_28_H_22_F_3_N_7_O	Approved; investigational	-9.7
14	DB12323	C_27_H_21_F_3_N_8_O	Investigational	-9.7
15	DB12719	C_25_H_24_F_2_N_2_O_3_	Investigational	-9.7
16	DB11791	C_23_H_17_FN_6_O	Approved; investigational	-9.6
17	DB11799	C_21_H_18_F_3_N_3_O_5_	Approved; investigational	-9.6
18	DB11977	C_33_H_37_F_2_N_7_O_4_	Investigational	-9.6
19	DB13648	C_44_H_50_N_4_O_2_	Experimental	-9.6
20	DB00820	C_22_H_19_N_3_O_4_	Approved; investigational	-9.5
21	DB01761	C_28_H_29_F_3_N_6_	Experimental	-9.5
22	DB04016	C_40_H_35_N_2_O_6_P	Experimental	-9.5
23	DB06888	C_22_H_21_N_5_O_3_	Experimental	-9.5
24	DB12200	C_23_H_19_N_3_O_2_	Investigational	-9.5
25	DB13109	C_25_H_28_N_8_O_3_	Investigational	-9.5
26	DB01200	C_32_H_40_BrN_5_O_5_	Approved; investigational	-9.4
27	DB01419	C_30_H_26_F_6_N_4_O_2_	Approved	-9.4
28	DB04330	C_29_H_19_Cl_2_N_3_O_6_S	Experimental	-9.4
29	DB06630	C_30_H_25_F_10_NO_3_	Investigational	-9.4
30	DB07020	C_20_H_14_N_6_O_2_	Experimental	-9.4

The top 30 compounds were distributed among four different groups of compounds. Among these, we selected the compounds with the best docking energy. For the “approved” type, we selected two compounds. Next, we analyzed the interactions of the compounds C_33_H_35_N_5_O_5_ (DB00696, generic name: ergotamine), C_30_H_26_F_6_N_4_O_2_ (DB01419, generic name: antrafenine), C_33_H_37_N_5_O_5_ (DB00320, generic name: dihydroergotamine), C_21_H_16_N_6_ (DB08450, generic name: N-1H-indazol-5-yl-2-(6-methylpyridin-2-yl) quinazolin-4-amine), and C_32_H_18_N_8_ (DB12983, generic name: phthalocyanine) with M^pro^.

**FIGURE 5:**
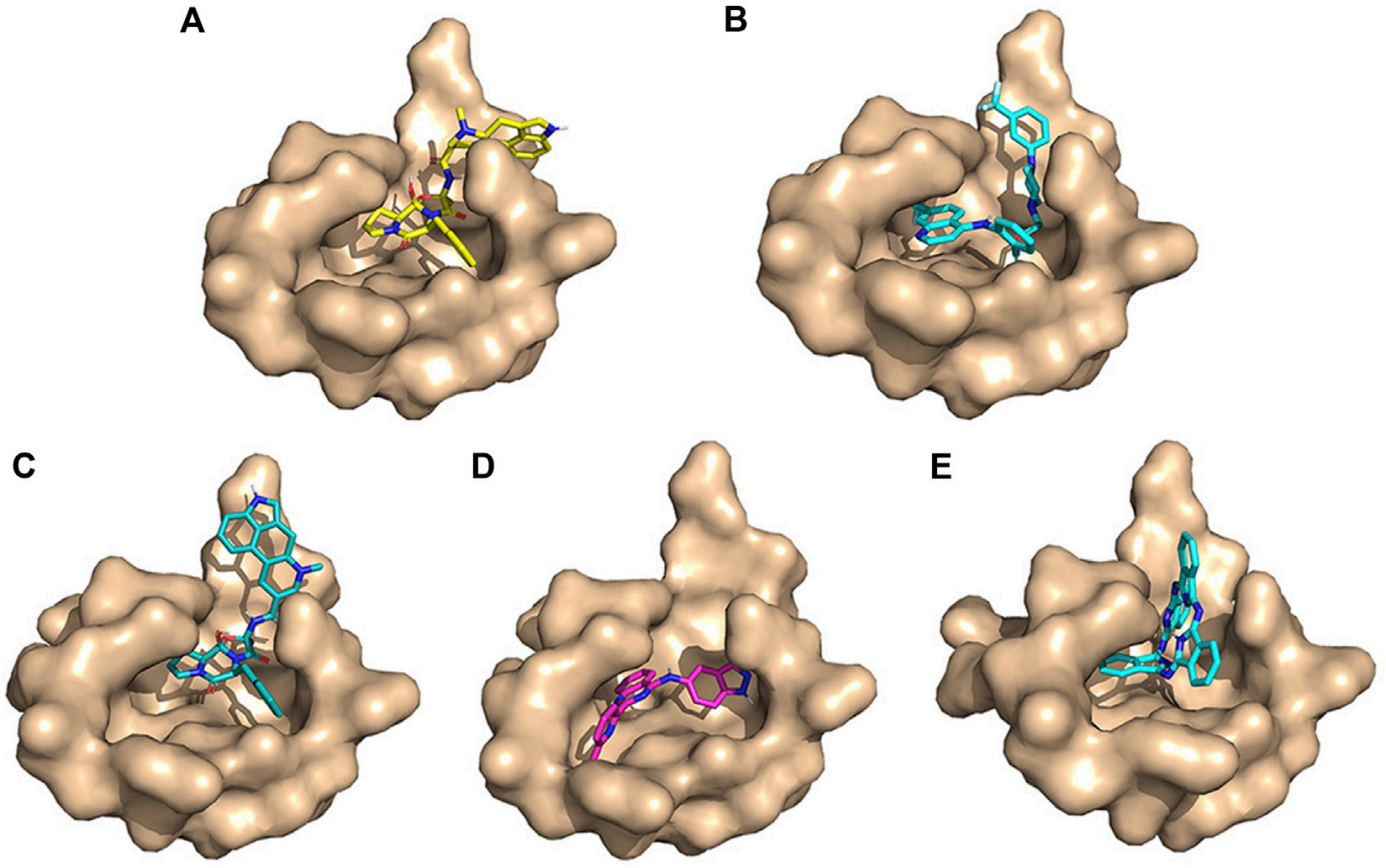
Conformation diagrams of these compounds. **(A)** Ergotamine; **(B)** antrafenine; **(C)** dihydroergotamine; **(D)** N-1H-indazol-5-yl-2-(6-methylpyridin-2-yl)quinazolin-4-amine; and **(E)** phthalocyanine.

**FIGURE 6:**
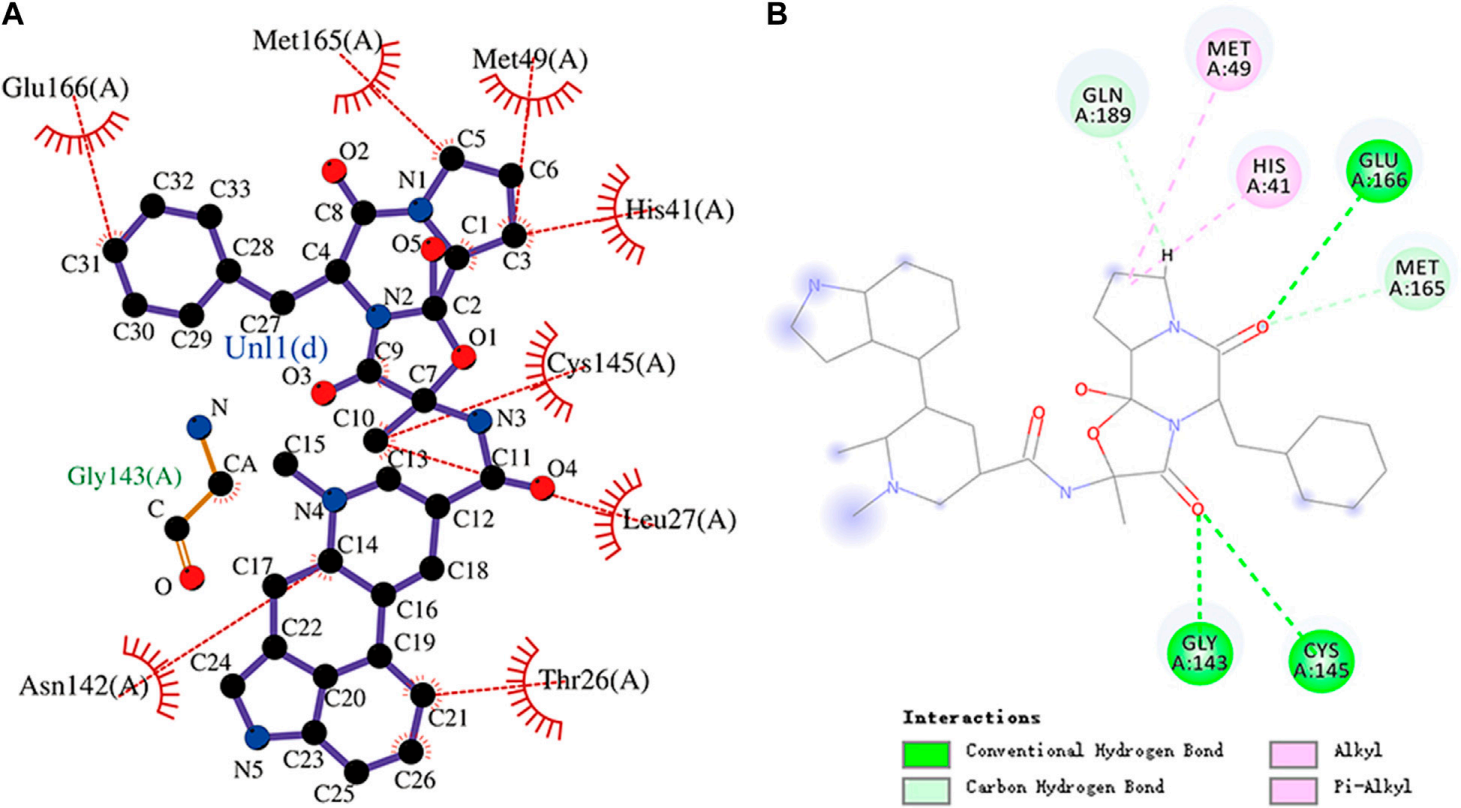
**(A)** Hydrophobic interaction between M^pro^ and ergotamine and **(B)** two-dimensional plot of ergotamine interaction with the amino acid residues.

**FIGURE 7:**
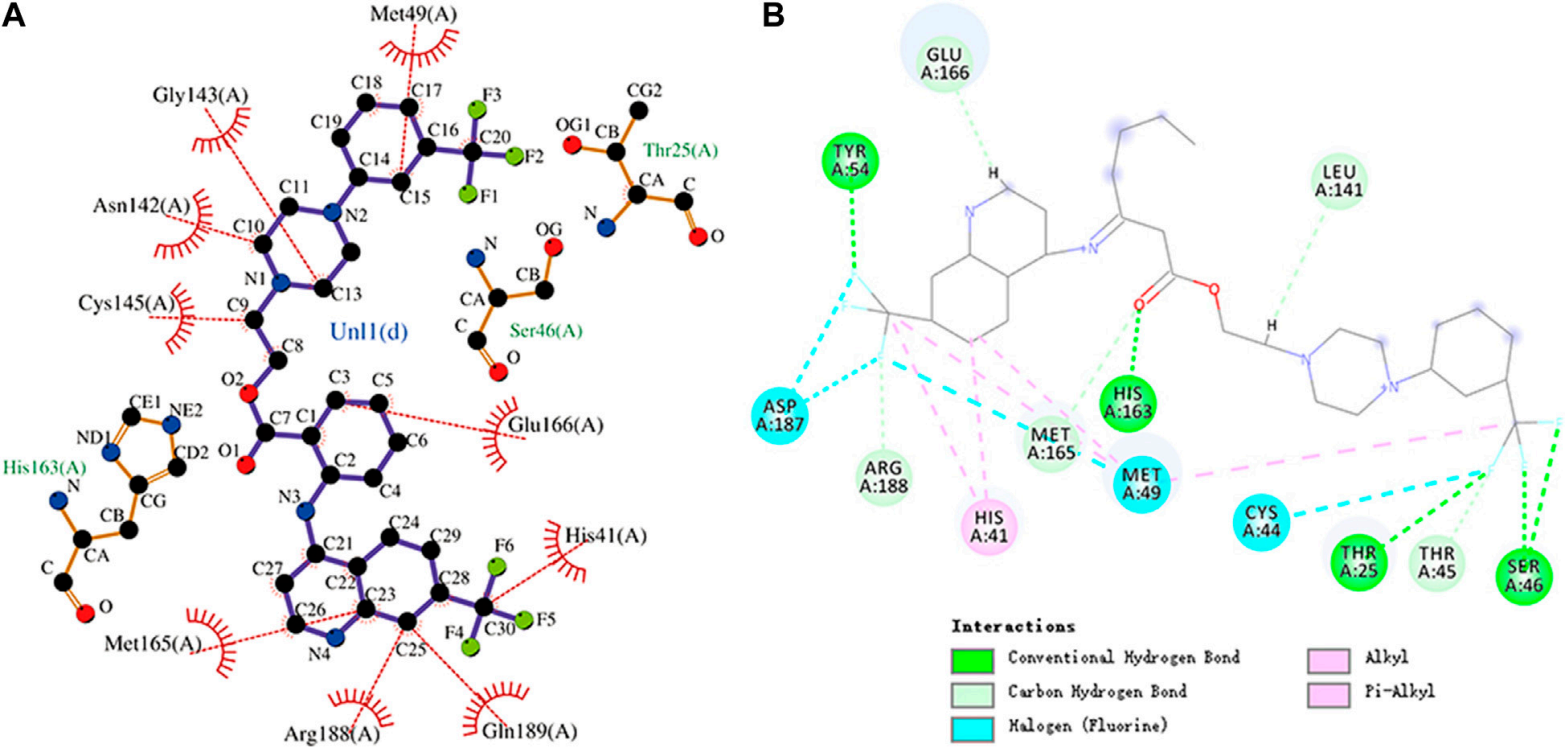
**(A)** Hydrophobic interaction between M^pro^ and antrafenine and **(B)** two-dimensional plot of antrafenine interaction with the amino acid residues.

**FIGURE 8:**
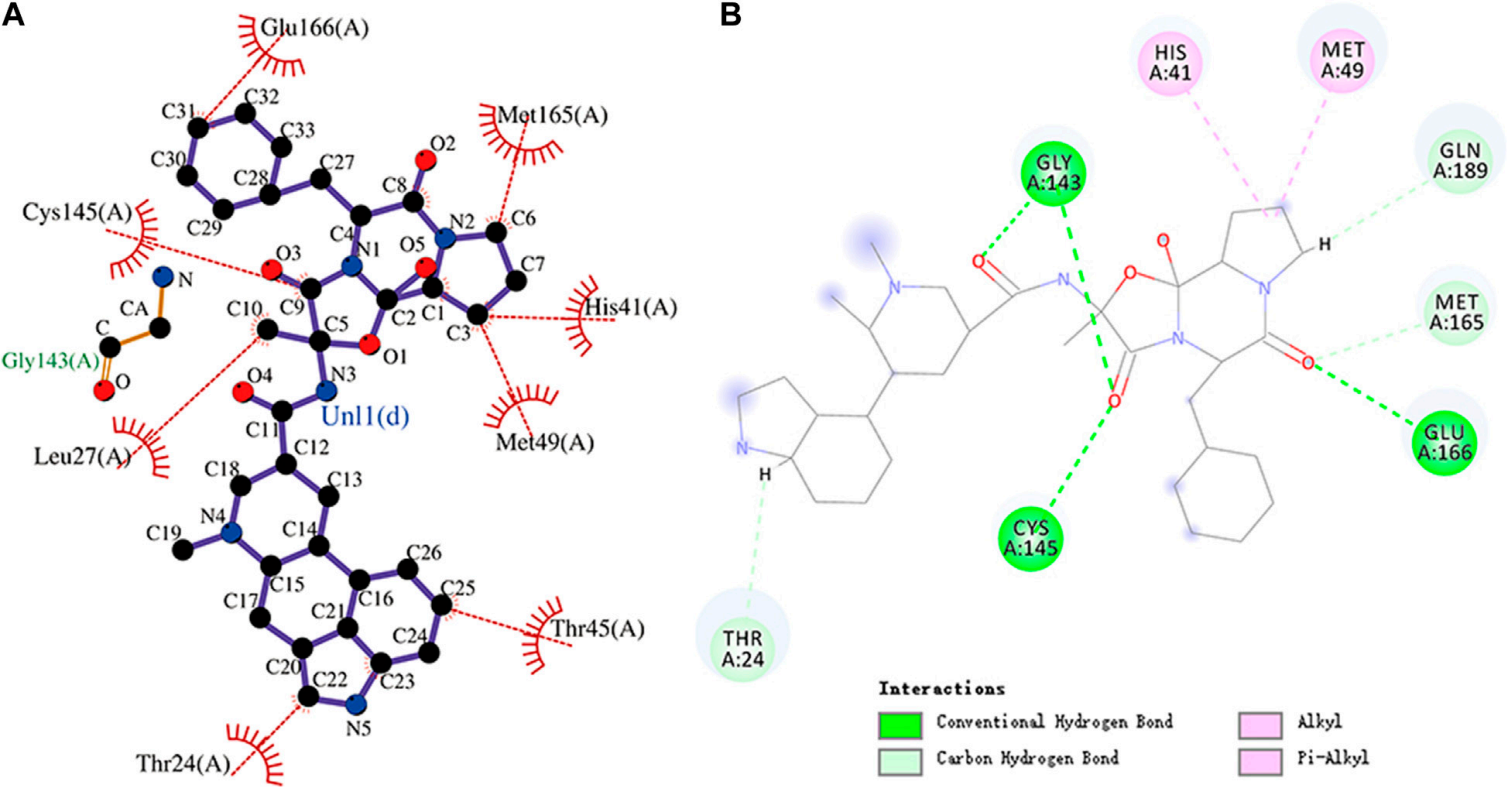
**(A)** Hydrophobic interaction between M^pro^ and dihydroergotamine and **(B)** two-dimensional plot of dihydroergotamine interaction with the amino acid residues.

**FIGURE 9:**
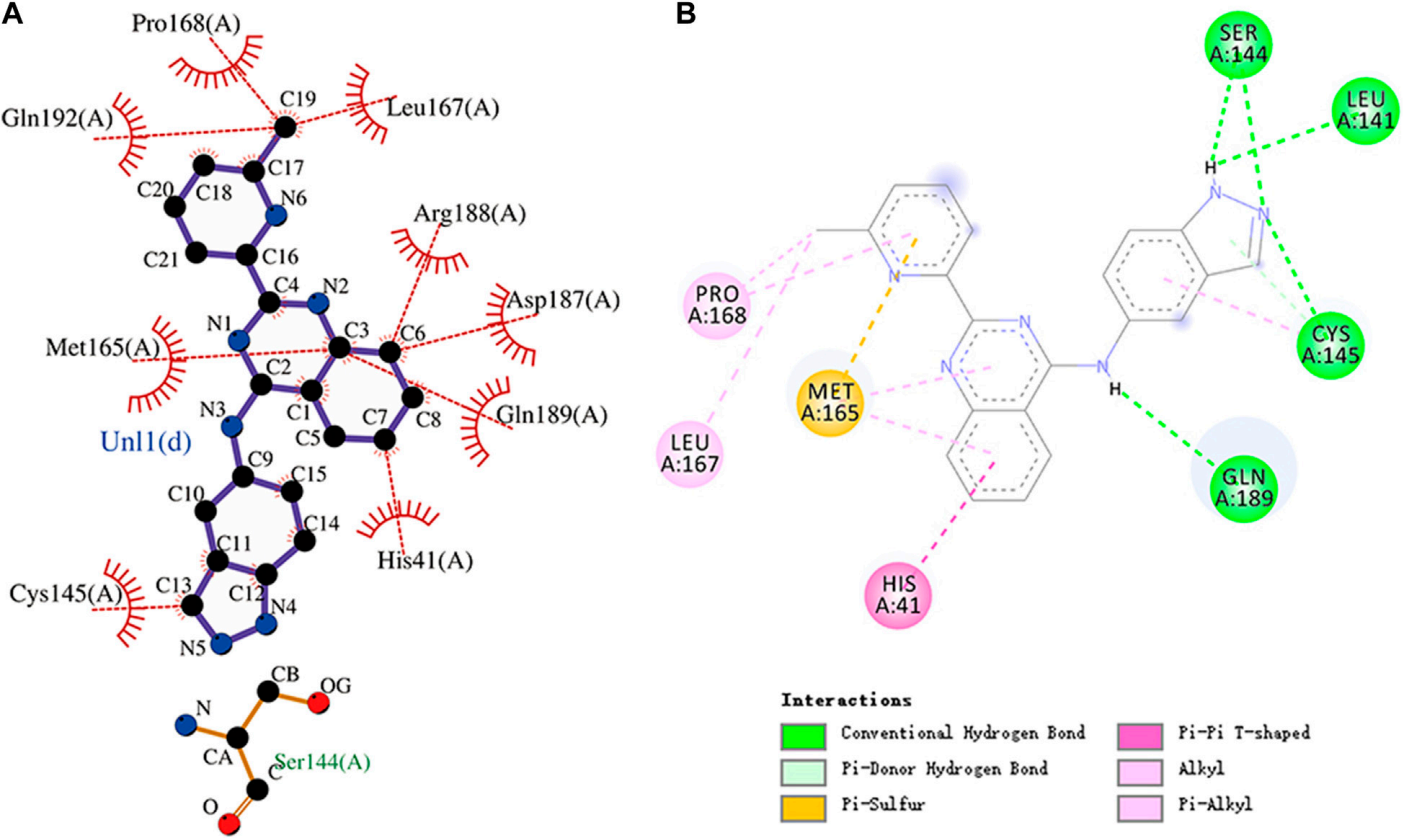
**(A)** Hydrophobic interaction between M^pro^ and N-1H-indazol-5-yl-2-(6-methylpyridin-2-yl)quinazolin-4-amine and **(B)** two-dimensional plot of N-1H-indazol-5-yl-2-(6-methylpyridin-2-yl)quinazolin-4-amine interaction with the amino acid residues.

**FIGURE 10:**
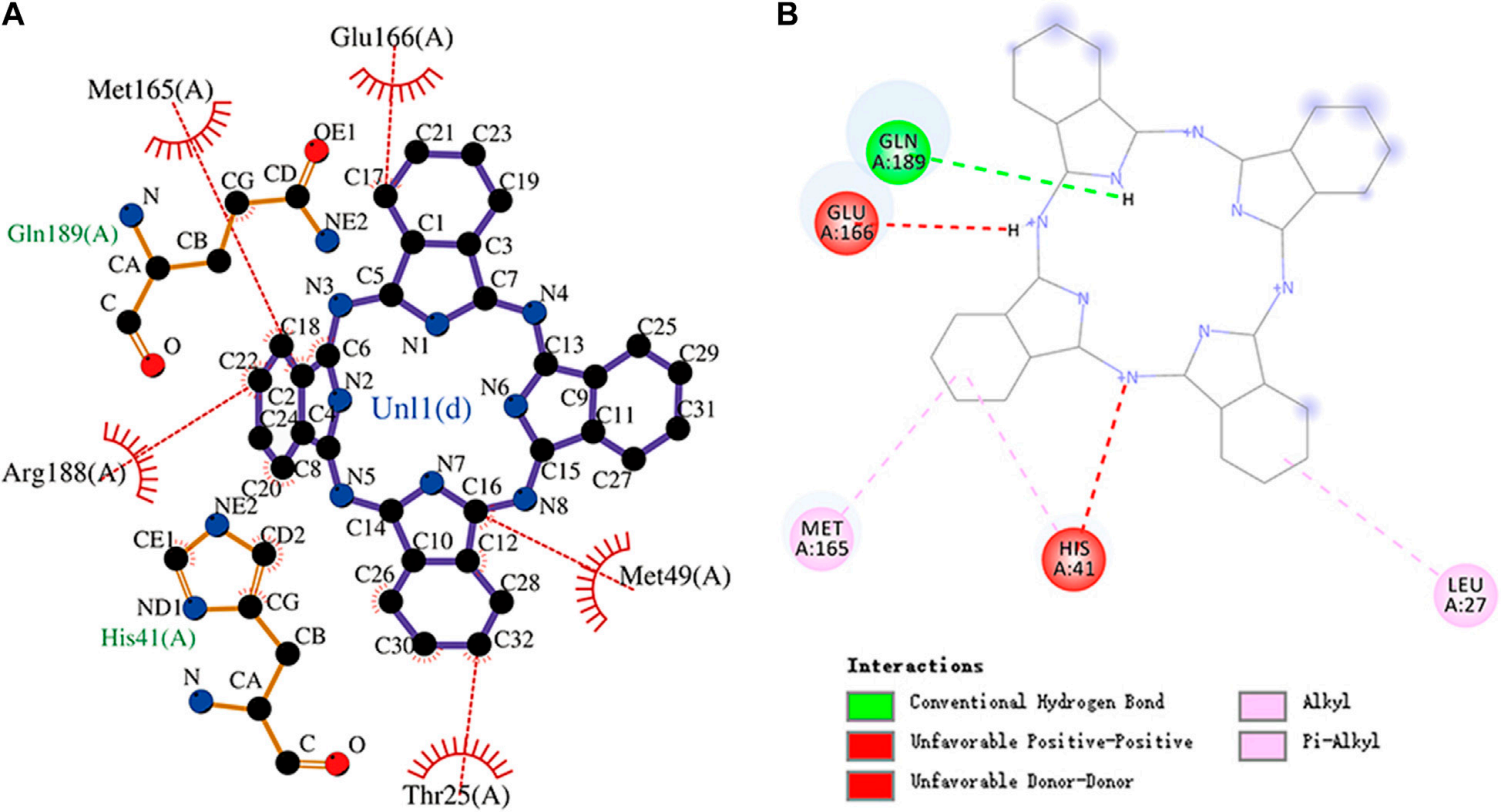
**(A)** Hydrophobic interaction between M^pro^ and phthalocyanine and **(B)** two-dimensional plot of phthalocyanine interaction with the amino acid residues.

The conformation diagrams of these compounds are displayed in Figure 5. The interactions of the M^pro^ protease with each of the five molecules are shown in Figures 6–10. Hydrophobic interactions were visualized using LIGPLOT (Laskowski and Swindells, 2011) Figure 7. Other interactions, including conventional hydrogen bond, carbon–hydrogen bond, pi–donor hydrogen bond, alkyl, pi–alkyl, halogen (fluorine), and pi–pi t-shaped, were visualized using Discovery Studio Visualizer. The interactions of residues with their respective ligands are shown in Supplementary Table S2.

### 3.2 Molecular dynamics simulation

We performed 100 ns MD simulations for each of the five compounds and N3 inhibitors in complex with M^pro^. As shown in Figure 8, complex N3–M^pro^ trajectory stabilized around 20 ns, N-1H-indazol-5-yl-2-(6-methylpyridin-2-yl)quinazolin-4-amine–M^pro^ stabilized around 25 ns, phthalocyanine–M^pro^ stabilized around 35 ns, antrafenine–M^pro^ stabilized around 25 ns, ergotamine–M^pro^ stabilized around 25 ns, and dihydroergotamine–M^pro^ stabilized around 25 ns The results showed that the RMSD of the five complexes in the MD trajectory interval (35–50 ns) fluctuated from 2.47 to 3.59 Å for dihydroergotamine Figure 9, 2.71–3.71 Å for ergotamine, 2.43–4.75 Å for phthalocyanine, 2.99–4.56 Å for antrafenine, 2.25–3.40 Å for N-1H-indazol-5-yl-2-(6-methylpyridin-2-yl)quinazolin-4-amine, and 1.91–3.33 Å for the N3 inhibitor and M^pro^ complex. The smaller the RMSD value, the smaller the fluctuation of the complex structure. Compared with N3 inhibitors, the RMSD of the five molecule–M^pro^ complexes have little difference Figure 10.

**FIGURE 11:**
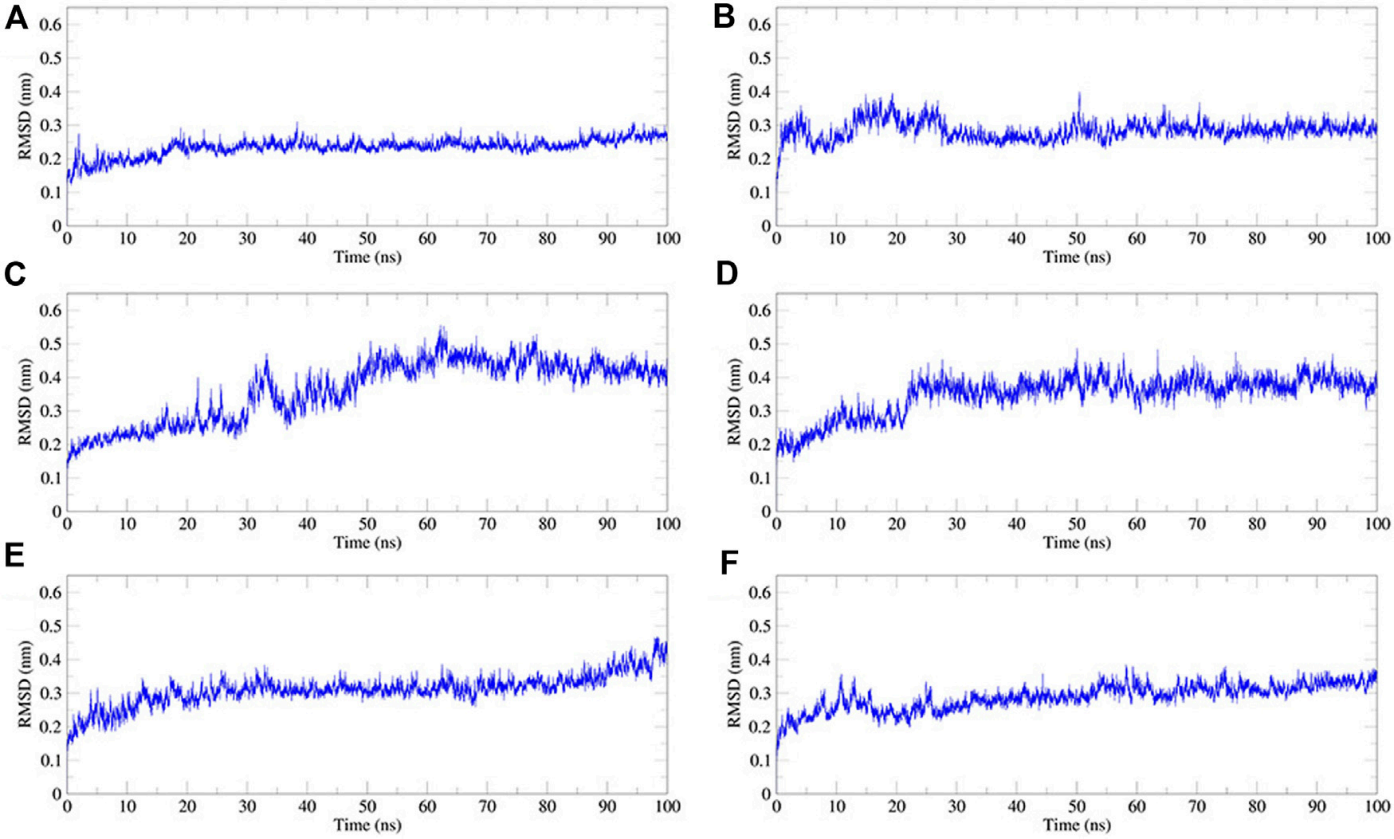
Plot of root mean square deviation (RMSD) values, during 100 ns MD simulation of compound–M^pro^ complexes. **(A)** N3–M^pro^ RMSD; **(B)** N-1H-indazol-5-yl-2-(6-methylpyridin-2-yl)quinazolin-4-amine–M^pro^ RMSD; **(C)** phthalocyanine–M^pro^ RMSD; **(D)** antrafenine–-M^pro^ RMSD **(E)** ergotamine–M^pro^ RMSD; and **(F)** dihydroergotamine–M^pro^ RMSD.

**FIGURE 12:**
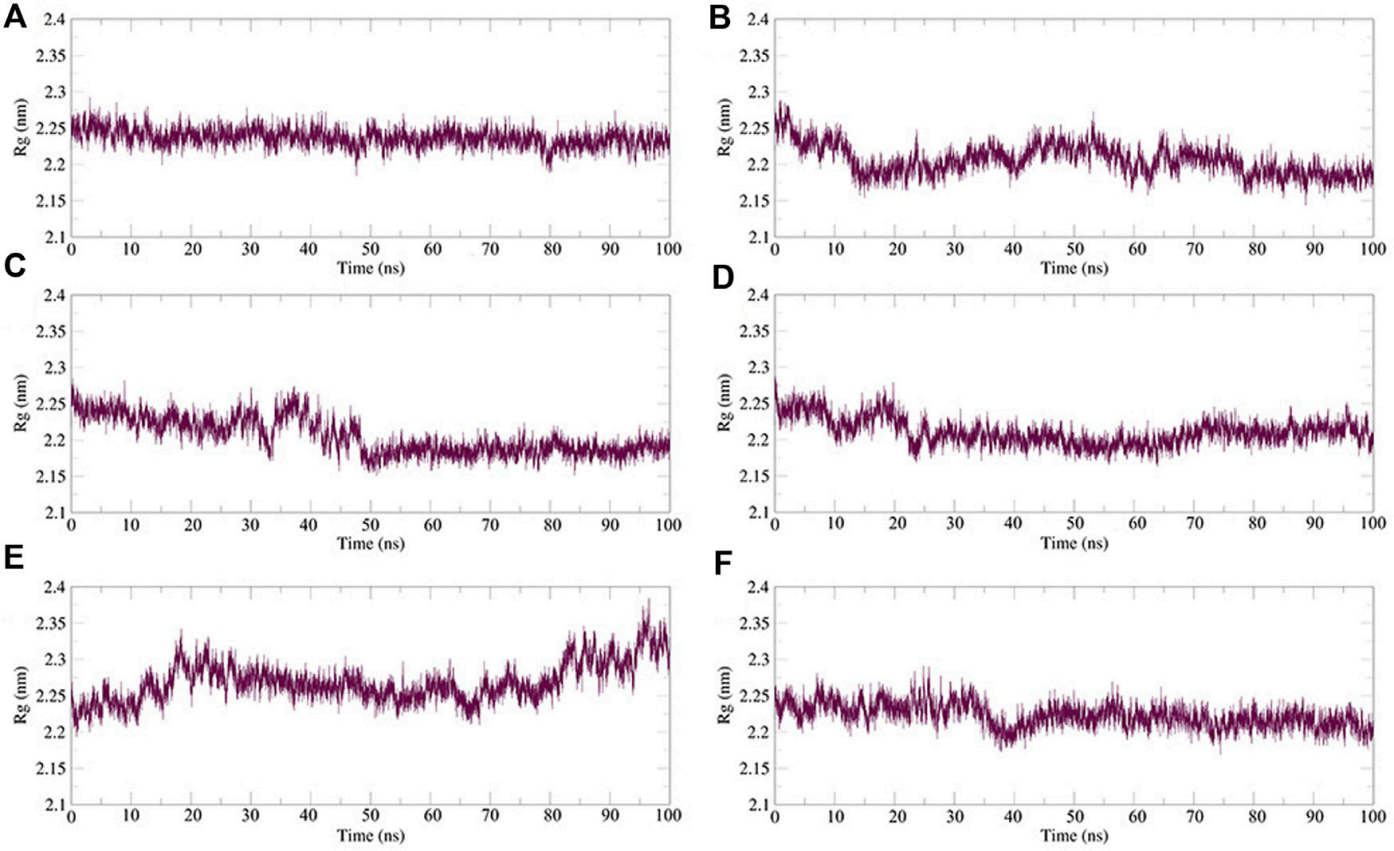
Plot of radius of gyration (Rg) values, during 100 ns MD simulation of compound–M^pro^ complexes. **(A)** N3–M^pro^ Rg; **(B)** N-1H-indazol-5-yl-2-(6-methylpyridin-2-yl)quinazolin-4-amine–M^pro^ Rg; **(C)** phthalocyanine–M^pro^ Rg; **(D)** antrafenine–M^pro^ Rg; **(E)** ergotamine–M^pro^ Rg; and **(F)** dihydroergotamine–M^pro^ Rg.

Rg is an important indicator for evaluating the compactness of the docking architecture. The smaller the cyclotron radius, the better the compactness Figure 11, and hence, the more stable the protein structure. The Rg results of the five molecules and N3 inhibitors in the complex with M^pro^ are shown in Figure 12. The average Rg of the N3–M^pro^ complex was about 22.5 Å. The average Rg of the five complexes showed little difference and was lower than that of the N3–M^pro^ complex except for ergotamine–M^pro^ complex. The average Rg of the drug dihydroergotamine–M^pro^, phthalocyanine–M^pro^, N-1H-indazol-5-yl-2-(6-methylpyridin-2-yl) quinazolin-4-amine–M^pro^, and antrafenine–M^pro^ complexes were all about 22 Å. The average Rg of the ergotamine–M^pro^ complex was about the highest (22.8 Å). This shows that the results of the Rg analysis are consistent with the results of the RMSD trajectory analysis.

**FIGURE 13:**
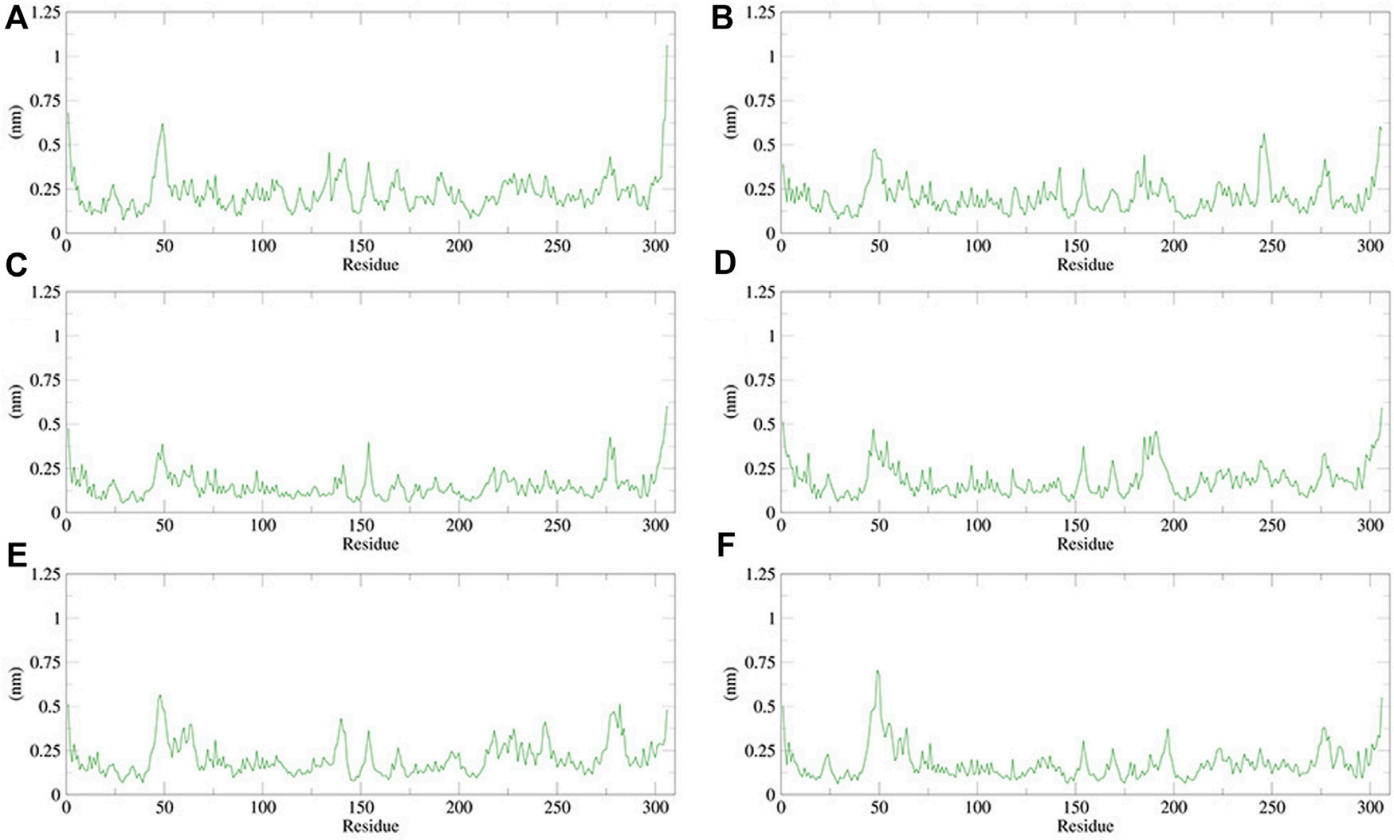
Plot of root mean square fluctuations (RMSF) values, during 100 ns MD simulation of compound–M^pro^ complexes. **(A)** N3–M^pro^ RMSF; **(B)** N-1H-indazol-5-yl-2-(6-methylpyridin-2-yl)quinazolin-4-amine–M^pro^ RMSF; **(C)** phthalocyanine–M^pro^ RMSF; **(D)** antrafenine–M^pro^ RMSF; **(E)** ergotamine–M^pro^ RMSF; and **(F)** dihydroergotamine–M^pro^ RMSF.

The RMSF can be used to observe how individual amino acids fluctuate during the simulation. It is possible to compare the effects of different small-molecule ligands on the spatial structural fluctuations of proteins by calculating the RMSF value. The smaller the value of RMSF, the smaller the disturbance of the small-molecule ligand to the protein, and therefore the stronger the stability of the complex. We calculated the RMSF value for each of the five small molecules and N3 inhibitors bound to M^pro^. The calculated values are shown in Figure 13. The RMSF results showed that the average RMSF value of M^pro^ bound to dihydroergotamine was 1.75 Å, which indicates less fluctuation in the complex structure. However, the residues Met49 (7.07 Å), Tyr54 (3.67 Å), Arg188 (2.30 Å), and Thr24 (2.30 Å) showed a slight fluctuation in the dihydroergotamine–M^pro^ complex during the simulation. Thus, from the perspective of RMSF, the stability order of the complex formed with the main protease is dihydroergotamine, antrafenine, ergotamine, N-1H-indazol-5-yl-2-(6-methylpyridin-2-yl) quinazolin-4-amine, and phthalocyanine.

**FIGURE 14:**
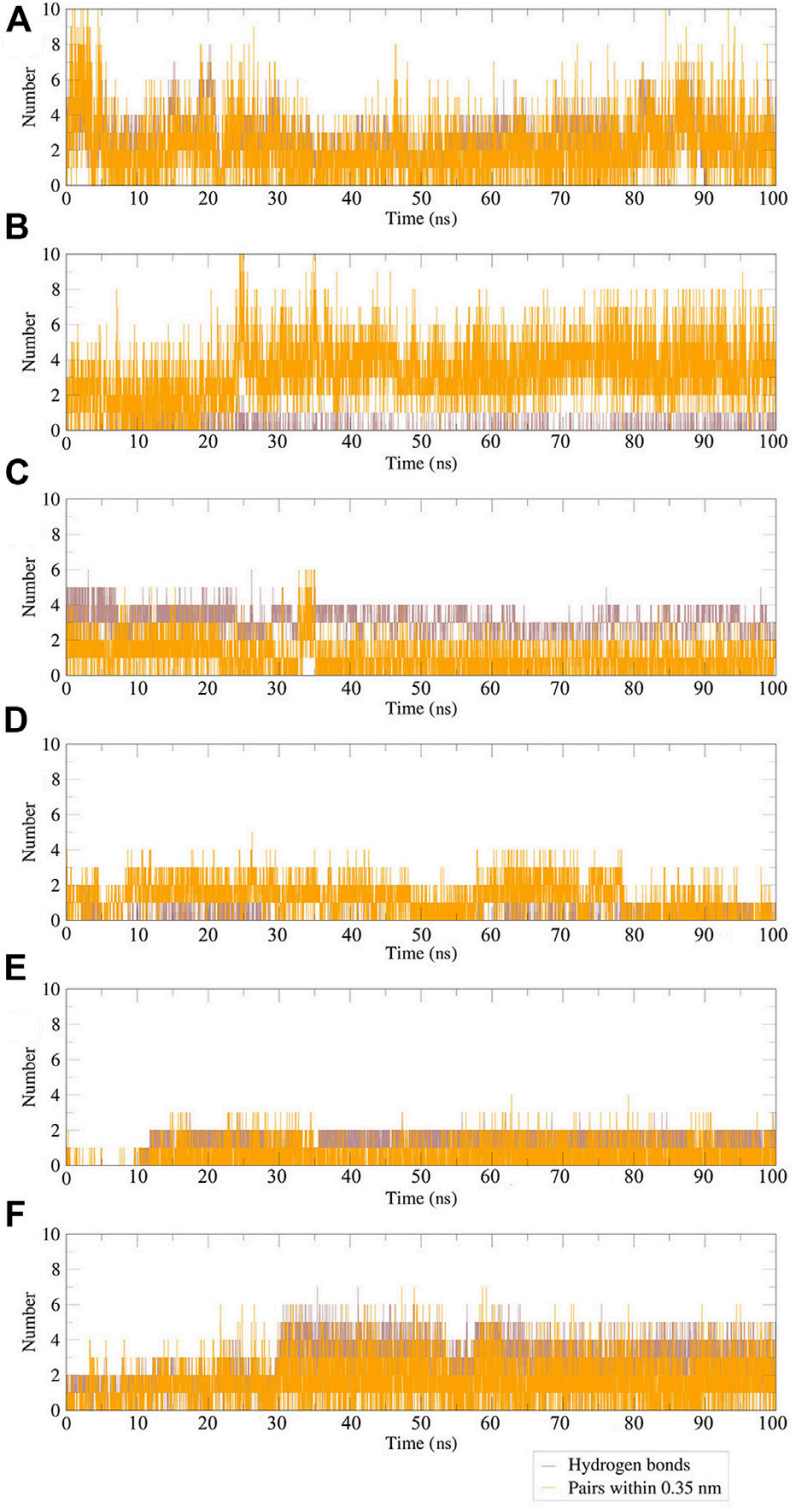
Plot of hydrogen bonds in compound–M^pro^ complexes during 100 ns MD simulation. **(A)** N3–M^pro^; **(B)** N-1H-indazol-5-yl-2-(6-methylpyridin-2-yl)quinazolin-4-amine–M^pro^; **(C)** antrafenine–M^pro^; **(D)** ergotamine–M^pro^; **(E)** dihydroergotamine–M^pro^; **(F)** phthalocyanine–M^pro^.

Hydrogen bonds between the ligand and key residues of the main protease were investigated using 100 ns MD simulations as shown in Figure 14. During the 100 ns simulation, there were multiple hydrogen bonds between the five compounds and M^pro^. The results confirmed that the five compounds in the MD system had a strong inhibitory effect on M^pro^, and there was a good binding effect between the compounds and M^pro^ in the pocket of M^pro^.

### 3.3 Pharmacodynamic properties

The results of bioactivity and ADMET are shown in Supplementary Tables S3, S4, respectively.

Molinspiration Cheminformatics can predict the GPCR ligand, ion channel modulator, kinase inhibitor, nuclear receptor ligand, protease inhibitor, and enzyme inhibitor values of compounds to evaluate their biological activities. According to reports, a bioactivity score of -5.0–0.0 is considered moderately active, and a score of ≥0 is considered active (Mokhnache et al., 2019; Rahman et al., 2021). From the predicted results, it can be concluded that N-1H-indazol-5-yl-2-(6-methylpyridin-2-yl)quinazolin-4-amine (score 0.38) is an active enzyme inhibitor, and the other four compounds can be approximately considered active enzyme inhibitors.

The admetSAR can predict ADMET for pharmacodynamic studies of five compounds in the host. In Supplementary Table S4, parameters such as molecular weight, water solubility (logS), human intestinal absorption, blood–brain barrier, Caco-2 permeable, human oral bioavailability, and toxicity of the compounds were listed. The results showed that the solubility value of antrafenine was slightly lower than -4, while the values of other compounds were higher than -4. This indicated that the solubility of the five compounds was suitable (Rahman et al., 2021). None of the five compounds were carcinogenic. But it should be noted that phthalocyanine and N-1H-indazol-5-yl-2-(6-methylpyridin-2-yl)quinazolin-4-amine were shown to have AMES toxicity. Regarding drug-likeliness, only N-1H-indazol-5-yl-2-(6-methylpyridin-2-yl)quinazolin-4-amine met the requirements of the five rules, and other compounds were larger than the ideal molecular weight of 500. By comparison, it was concluded that the drug candidate order of the five compounds was ergotamine, dihydroergotamine, antrafenine, N-1H-indazol-5-yl-2-(6-methylpyridin-2-yl)quinazolin-4-amine, and phthalocyanine.

## 4 Discussion

The docking of small-molecule compounds to receptor binding sites and the estimation of the binding affinity of the complex are important components of the structure-based drug design process. AutoDock Vina is an open-source program for drug discovery, molecular docking, and virtual screening, which significantly improves the average accuracy of the binding mode predictions (Herowati and Widodo, 2014; Xiang et al., 2015).

Ergotamine is an *α*-1 selective adrenergic agonist that is commonly used in the treatment of migraine disorders. The binding energy of ergotamine and M^pro^ was -9.7 kcal/mol. Ergotamine forms hydrogen bonds with residues Gly143, Cys145, and Glu166, respectively. Glu166, Met165, Met49, His41, Cys145, Leu27, Thr26, and Asn142 residues and the hydrophobic groups of ergotamine can engage through hydrophobic interactions. Ergotamine has an alkyl interaction with residue Met49 and a pi–alkyl interaction with residue His41. The molecular docking representation of ergotamine with M^pro^ is shown in Figure 6.

The interaction energy between antrafenine and M^pro^ was -9.4 kcal/mol. Antrafenine forms hydrogen bonds with residues Thr25, Ser46, Tyr54, and His163, respectively. Met49, Glu166, His41, Gln189, Arg188, Met165, Cys145, Asn142, and Gly143 residues and antrafenine can engage through hydrophobic interactions. There were also alkyl, pi–alkyl, and halogen (fluorine) interactions between antrafenine and residues. The molecular docking representation of antrafenine with M^pro^ is shown in. Moreover, antrafenine is a piperazine derivative drug, which exhibits analgesic and anti-inflammatory effects similar to naproxen.

The interaction energy between dihydroergotamine and M^pro^ was -9.9 kcal/mol. Dihydroergotamine forms hydrogen bonds with residues Gly143, Cys145, and Glu166, respectively. Moreover, dihydroergotamine is stabilized by the interaction with M^pro^ through hydrophobic interactions, involving Glu166, Leu27, Cys145, Thr24, Thr45, Met49, His41, and Met165 residues. There were also carbon–hydrogen bond, alkyl, and pi–alkyl interactions between dihydroergotamine and residues. The molecular docking representation of dihydroergotamine with M^pro^ is shown in .

N-1H-indazol-5-yl-2-(6-methylpyridin-2-yl)quinazolin-4-amine is an experimental drug molecule. The binding energy of N-1H-indazol-5-yl-2-(6-methylpyridin-2-yl)quinazolin-4-amine and M^pro^ was -9.8 kcal/mol. N-1H-indazol-5-yl-2-(6-methylpyridin -2-yl)quinazolin-4-amine forms hydrogen bonds with residues Leu141, Ser144, Cys145, and Gln189, respectively. Gln192, Pro168, and Leu167 residues and the methyl of N-1H-indazol-5-yl-2-(6-methylpyridin-2-yl)quinazolin-4-amine can engage through hydrophobic interactions. Furthermore, Arg188, Asp187, Met165, Gln189, His41, and Cys145 residues can form hydrophobic interactions with N-1H-indazol-5-yl-2-(6-methylpyridin -2-yl)quinazolin-4-amine. There were also pi–donor hydrogen bond, alkyl, pi–alkyl, halogen (fluorine), and pi–pi T-shaped interactions between N-1H-indazol-5-yl-2-(6 -methylpyridin-2-yl)quinazolin-4-amine and residues. The molecular docking representation of N-1H-indazol-5-yl-2-(6-methylpyridin-2-yl)quinazolin-4-amine with M^pro^ is shown in.

Phthalocyanine is an 18-electron large conjugated system compound comprising four isoindole units. The center of the conjugated ring structure has a large cavity that can accommodate metal ions (such as iron, cobalt, and nickel). Phthalocyanine has the lowest binding energy value of -10.7 kcal/mol. Glu166, Met165, Arg188, His41, Met49, and Thr25 residues and the isoindole ring of phthalocyanine can engage through hydrophobic interactions. The carbonyl group of Gln189 (hydrogen bond acceptor) forms a hydrogen bond with the NH group of acting phthalocyanine (hydrogen bond donor). Phthalocyanine also has alkyl and pi–alkyl interactions with residues. However, it should be noted that phthalocyanine has unfavorable interactions with residues His41 and Glu166, respectively. The molecular docking representation of phthalocyanine with M^pro^ is shown in.

## 5 Conclusion

Molecular docking and molecular dynamics simulations have been widely used in drug screening and drug design. In this study, we present several exciting findings about SARS-CoV-2 M^pro^. The compounds analyzed in this study can be used as potential inhibitors of SARS-CoV-2 M^pro^: Ergotamine is an approved medication for the treatment of migraine disorders, and antrafenine is used as an anti-inflammatory and analgesic agent for the relief of mild-to-moderate pain. Furthermore, we have uncovered dihydroergotamine, N-1H-indazol-5-yl-2-(6-methylpyridin-2-yl) quinazolin-4-amine, and phthalocyanine, which may be developed as potential treatments against SARS-CoV-2 infections. Structural optimization and clinical trials are needed for these compounds to become strong drug candidates. At present, no biological experiments have been carried out in this study. However, through high-throughput molecular docking and molecular dynamics simulations, it was confirmed that these five compounds can form stable conformational structures with M^pro^ and have potential inhibitory effects on SARS-CoV-2. At the same time, this study provides research ideas and helps for drug designing and drug reusing for the treatment of SARS-CoV-2.

## Data Availability

Publicly available datasets were analyzed in this study. These data can be found at: the complex crystal structure of M^pro^ protein with an N3 inhibitor (PDB ID: 6lu7) was downloaded from the Protein Data Bank (http://www.rcsb.org). The drug molecule data set contains 8,820 molecules, with 3D structures, which is in the SDF format obtained from DrugBank (https://www.drugbank.ca/).
