# Supplementary material for: Screening of potential inhibitors targeting the main protease structure of SARS-CoV-2 via molecular docking
Source: Front Pharmacol. 2022 Oct 5;13:962863. doi: 10.3389/fphar.2022.962863 (PMC9579442; doi:10.3389/fphar.2022.962863)
Supplement: Supplementary file 1 [file Table2.DOCX]

Table S2. The interactions of residues with their respective ligands.

|  | **Ergotamine** | **Antrafenine** | **Dihydroergotamine** | **N-1H-indazol-5-yl-2-(6-methylpyridin-2-yl)quinazolin-4-amine** | **Phthalocyanine** |
| --- | --- | --- | --- | --- | --- |
| **Conventional Hydrogen Bond** | Gly143, Cys145, Glu166 | Thr25, Ser46, Tyr54, His163 | Gly143, Cys145, Glu166 | Leu141, Ser144, Cys145, Gln189 | Gln189 |
| **Carbon Hydrogen Bond** | Met165, Gln189 | Thr45, Leu141, Met165, Glu166, Arg188 | Thr24, Met165, Gln189 |  |  |
| **Pi-Donor Hydrogen Bond** |  |  |  | Cys145 |  |
| **Alkyl** | Met49 | Met49 | Met49 | Leu167, Pro168 | Leu27, Met165 |
| **Pi-Alkyl** | His41 | His41 | His41 | Met165, Cys145, Pro168 | His41 |
| **Halogen (Fluorine)** |  | Cys44, Met49, Asp187 |  | Met165 |  |
| **Pi-Sulfur** |  |  |  |  |  |
| **Pi-Pi T-shaped** |  |  |  | His41 |  |
| **Unfavorable Positive-Positive** |  |  |  |  | His41 |
| **Unfavorable Donor-Donor** |  |  |  |  | Glu166 |
