# Supplementary material for: Screening of potential inhibitors targeting the main protease structure of SARS-CoV-2 via molecular docking
Source: Front Pharmacol. 2022 Oct 5;13:962863. doi: 10.3389/fphar.2022.962863 (PMC9579442; doi:10.3389/fphar.2022.962863)
Supplement: Supplementary file 2 [file Table3.DOCX]

Table S3. Bioactivity prediction of five compounds by Molinspiration.

| **Bioactivity Scores** | **Ergotamine** | **Antrafenine** | **Dihydroergotamine** | **N-1H-indazol-5-yl-2-(6-methylpyridin-2-yl)quinazolin-4-amine** | **Phthalocyanine** |
| --- | --- | --- | --- | --- | --- |
| **GPCR ligand** | **0.73** | **0.05** | **0.61** | **0.34** | **-0.01** |
| **Ion channel modulator** | **-0.36** | **-0.27** | **-0.34** | **0.14** | **-0.23** |
| **Kinase inhibitor** | **-0.32** | **0.16** | **-0.30** | **0.93** | **0.10** |
| **Nuclear receptor ligand** | **-0.43** | **-0.19** | **-0.51** | **-0.03** | **-0.19** |
| **Protease inhibitor** | **0.20** | **-0.08** | **0.29** | **-0.17** | **-0.07** |
| **Enzyme inhibitor** | **-0.12** | **-0.15** | **-0.15** | **0.38** | **-0.04** |
