# Supplementary material for: Screening of potential inhibitors targeting the main protease structure of SARS-CoV-2 via molecular docking
Source: Front Pharmacol. 2022 Oct 5;13:962863. doi: 10.3389/fphar.2022.962863 (PMC9579442; doi:10.3389/fphar.2022.962863)
Supplement: Supplementary file 4 [file Table4.DOCX]

Table S4. Pharmacodynamic properties prediction via admetSAR web-service

|  | **Ergotamine** | **Antrafenine** | **Dihydroergotamine** | **N-1H-indazol-5-yl-2-(6-methylpyridin-2-yl)quinazolin-4-amine** | **Phthalocyanine** |
| --- | --- | --- | --- | --- | --- |
| **Molecular Weight** | 581.67 | 588.55 | 583.69 | 352.40 | 514.55 |
| **AlogP** | 1.99 | 7.00 | 2.08 | 4.62 | 6.77 |
| **Water solubility (logS)** | -2.679 | -4.397 | -2.844 | -2.615 | -3.584 |
| **Human Intestinal Absorption** | 0.9760 | 0.9873 | 0.9411 | 0.9969 | 0.9917 |
| **Blood Brain Barrier** | 0.9351 | 0.9946 | 0.9930 | 0.5360 | 0.9716 |
| **Caco-2 permeable** | 0.8399 | 0.8248 | 0.8370 | 0.3437 | 0.7573 |
| **Human oral bioavailability** | 0.9429 | 0.8143 | 0.9143 | 0.5571 | 0.8000 |
| **Ames test** | Non AMES toxic | Non AMES toxic | Non AMES toxic | AMES toxic | AMES toxic |
| **Carcinogenicity** | Non-carcinogens | Non-carcinogens | Non-carcinogens | Non-carcinogens | Non-carcinogens |
| **Rat acute toxicity (LD50, mol/kg)**  **Rule of Five** | 2.984  No | 2.7506  No | 2.9503  No | 2.3982  Yes | --  No |
